# Reducing Heart Failure Hospitalizations With Proactive Outpatient Diuretic Management: A Real-World Observational Study

**DOI:** 10.1016/j.jaccas.2026.109117

**Published:** 2026-09-16

**Authors:** Aqsa Iqbal, Sannie Sulley, Lee Anheuser, Faelynn Hammond, Chrystal Grant, Corrie Farris, Amy Glasco, Arslan Mirza

**Affiliations:** a Southern Illinois HealthCare, Carbondale, Illinois, USA; b Southern Illinois University School of Medicine, Springfield, Illinois, USA; c National Healthy Start Association, Washington, DC, USA; d Prairie Heart Institute, Carbondale, Illinois, USA

**Keywords:** diuretic optimization, heart failure, hospitalization, quality improvement

## Abstract

**Background:**

Recurrent congestion remains a major driver of heart failure (HF)–related acute care utilization despite guideline-directed medical therapy.

**Project Rationale:**

The Congestive Heart Failure Navigator program was developed to support early recognition of worsening HF and proactive HF optimization. This study evaluated whether higher outpatient diuretic optimization intensity was associated with reduced HF hospitalizations.

**Project Summary:**

This retrospective quality improvement study evaluated patients enrolled in the Congestive Heart Failure Navigator program during 2025. Temporal associations between prior-month oral diuretic optimization and HF hospitalizations were analyzed using regression models. The program managed a mean of 54 patients monthly, with hospitalization rates remaining low (0-9.4 per 100 patients). Higher outpatient diuretic optimization intensity was associated with fewer subsequent HF hospitalizations (incidence rate ratio: ≈0.94; *P* ≈ 0.046). In a pre-post analysis (n = 38), HF hospitalizations declined from 34.2% to 18.4%.

**Take-Home Message:**

Higher-intensity outpatient oral diuretic optimization may represent a scalable early congestion management strategy associated with reduced HF hospitalizations.

## Visual Summary

**Figure:**
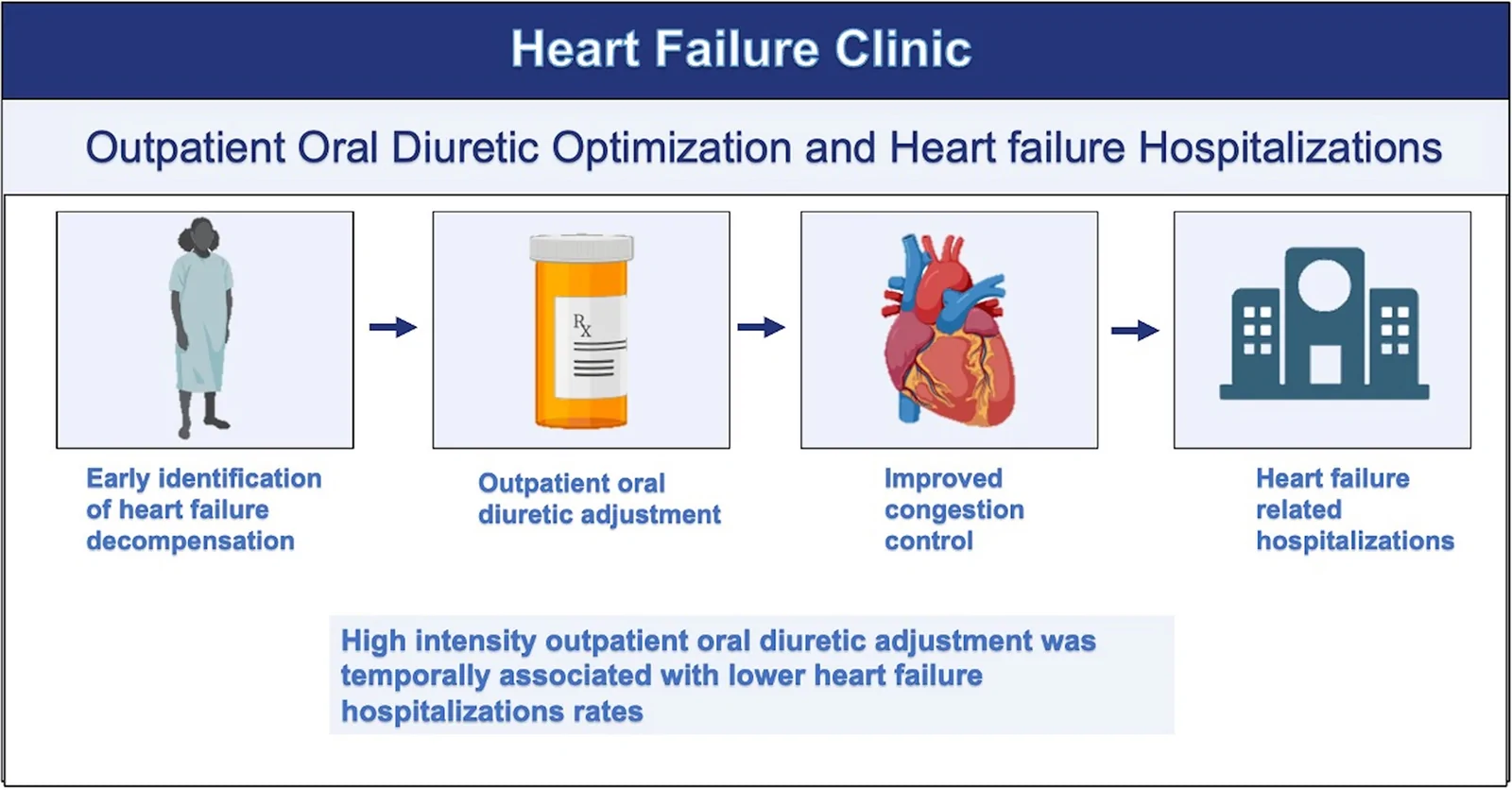

Heart failure (HF) remains a leading cause of emergency department (ED) visits, hospitalizations, and health care expenditures. In the United States, HF accounts for approximately 1.2 million hospitalizations annually and affects an estimated 6.7 million adults.^1^^,^^2^ Despite advances in guideline-directed medical therapy (GDMT), recurrent congestion continues to drive HF decompensation and hospitalization.^3^ Structured outpatient HF programs improve outcomes and reduce HF-related hospitalizations.^4^^,^^5^

Recognizing outpatient worsening HF as a key therapeutic window, early optimization of diuretic therapy may prevent progression from congestion to decompensation. Flexible symptom- and weight-guided loop diuretic adjustment has improved quality of life and may reduce HF hospitalizations.^6^ However, less is known about how to quantify the intensity of real-world outpatient congestion management—particularly oral diuretic optimization over time—and its relationship with HF-specific acute care utilization. Therefore, we evaluated whether higher outpatient oral diuretic optimization intensity within a structured HF program was associated with reduced HF-related acute care utilization over time.

## Project Rationale

Worsening congestion is a major driver of HF hospitalization and may be detectable before acute decompensation occurs. We evaluated a high-intensity outpatient HF program emphasizing early oral diuretic optimization for evolving congestion and its association with HF-related hospitalization reduction.

## Project Description

### Study design and setting

We performed a retrospective observational quality improvement study evaluating associations between outpatient oral diuretic optimization intensity and HF-related acute care utilization among patients enrolled in the Congestive Heart Failure (CHF) Navigator program during 2025. The program was developed several years earlier in response to recurrent HF admissions and the need for earlier outpatient recognition of worsening congestion after discharge. Institutional efforts aimed to maintain HF readmission rates near 21%, although increased utilization highlighted the need for structured outpatient monitoring.

The study was reviewed by the Southern Illinois Healthcare Institutional Review Board and deemed exempt, with a waiver of informed consent. Program-level and patient-level data were collected longitudinally. Because the program predated the analytical period, complete baseline institutional data were not consistently available. Therefore, analyses focused on 2025 program activity and included a patient-level pre-post analysis among patients with complete data. Given the observational design, analyses evaluated temporal associations rather than causality.

### CHF Navigator program description

The CHF Navigator program is a high-intensity outpatient HF management model focused on early recognition of worsening congestion and timely outpatient intervention. Because many patients did not consistently monitor daily weights or recognize early congestion symptoms, the program incorporated HF education, longitudinal follow-up, and home scale monitoring. Patients were followed through clinic visits and telephone encounters performed by CHF Navigator nursing staff and HF clinic personnel in collaboration with cardiology clinicians.

Management emphasized proactive outpatient oral diuretic optimization based on symptoms, weight gain, edema, dyspnea, renal function, electrolytes, blood pressure, and clinician judgment. Nursing protocols allowed patients with worsening congestion, including weight gain of >3 lb in 1 day or >5 lb in 1 week with symptoms, to receive 1 additional daily oral diuretic dose for up to 3 days. Patients with weight gain >10 lb were scheduled for urgent clinic evaluation by a provider or registered nurse depending on availability. Patients with persistent congestion despite oral therapy could receive outpatient intravenous (IV) diuretics, typically furosemide 80 mg IV dose or bumetanide 2 mg IV dose, with the goal of avoiding hospitalization.

### Measures of outpatient care intensity

Monthly program-level data included enrolled patients, HF education encounters, oral diuretic medication changes, IV diuretic administrations, and HF clinic visits. To account for variation in enrollment, all outpatient activity measures were standardized per 100 enrolled patients per month to reflect care intensity over time.

## Project Deliverables

### Outcomes

Primary outcomes were HF-related acute care utilization, defined as HF hospitalizations and HF-related ED visits, measured monthly and standardized per 100 enrolled patients. Absolute monthly counts described program volume, whereas standardized rates were used in figures and regression models to account for variation in monthly enrollment. Events were identified from administrative records and classified as HF related when HF was documented as the primary reason for presentation or admission.

### Statistical analysis

Descriptive statistics summarized outpatient care intensity and HF-related utilization over time. Poisson and negative binomial regression models were used to evaluate temporal trends in HF hospitalizations and ED visits, with enrollment included as an offset. Exploratory analyses assessed associations between oral diuretic optimization intensity and HF hospitalizations using both same-month and lagged models, with oral diuretic activity in month *t − 1* evaluated in relation to HF hospitalization rates in month *t*. A secondary patient-level pre-post analysis was conducted among patients with complete 3-month pre- and post-enrollment data to compare the proportion experiencing ≥1 HF hospitalizations before vs after enrollment using paired analyses. A 2-sided *P* value <0.05 was considered statistically significant.

## Project Outcome, Impact, and Future Direction

### Outpatient care intensity and diuretic optimization

Monthly enrollment ranged from approximately 32 to 67 patients (Table 1). Outpatient engagement remained consistent, with approximately 30 education encounters and 26 oral diuretic adjustments per month. Education encounters ranged from approximately 28 to 92 per 100 enrolled patients during several months, whereas oral diuretic optimization ranged from approximately 25 to 100 events.

**Table 1:** Characteristics of Outpatient HF Program and Acute Care Utilization

Program Reach	
Enrolled patients per month, mean (range)	54 (32-67)
Outpatient Care Intensity (Monthly Standardized Rates per 100 Enrolled Patients)	
Education encounters, mean (range)	56.2 (27.6-92.4)
Oral diuretic medication changes, mean (range)	51.2 (24.6-100.0)
IV diuretic administrations, mean (range)	11.2 (5.2-20.8)
HF-Related Acute Care Utilization (Monthly Standardized Rates per 100 Enrolled Patients)	
HF hospitalizations, mean (range)	3.2 (0-9.4)
HF-related emergency department visits, mean (range)	2.0 (0-17.3)
Patient-Level Pre-Post Analysis	
Patients with ≥1 HF hospitalization in 3 mo pre-enrollment	34.2%
Patients with ≥1 HF hospitalization in 3 mo post-enrollment	18.4%

Values are reported as monthly means and ranges standardized per 100 enrolled patients unless otherwise specified.

HF = heart failure; IV = intravenous.

### HF-related acute care utilization over time

HF-related acute care utilization remained low despite increasing program enrollment. Monthly HF hospitalization rates ranged from 0 to approximately 9.4 events per 100 enrolled patients, with multiple months demonstrating no hospitalizations. HF-related ED visits were infrequent overall, with 1 isolated monthly spike was observed. Regression analyses demonstrated no statistically significant temporal increase in HF hospitalization or ED visit rates (Figure 1).

**Figure 1:**
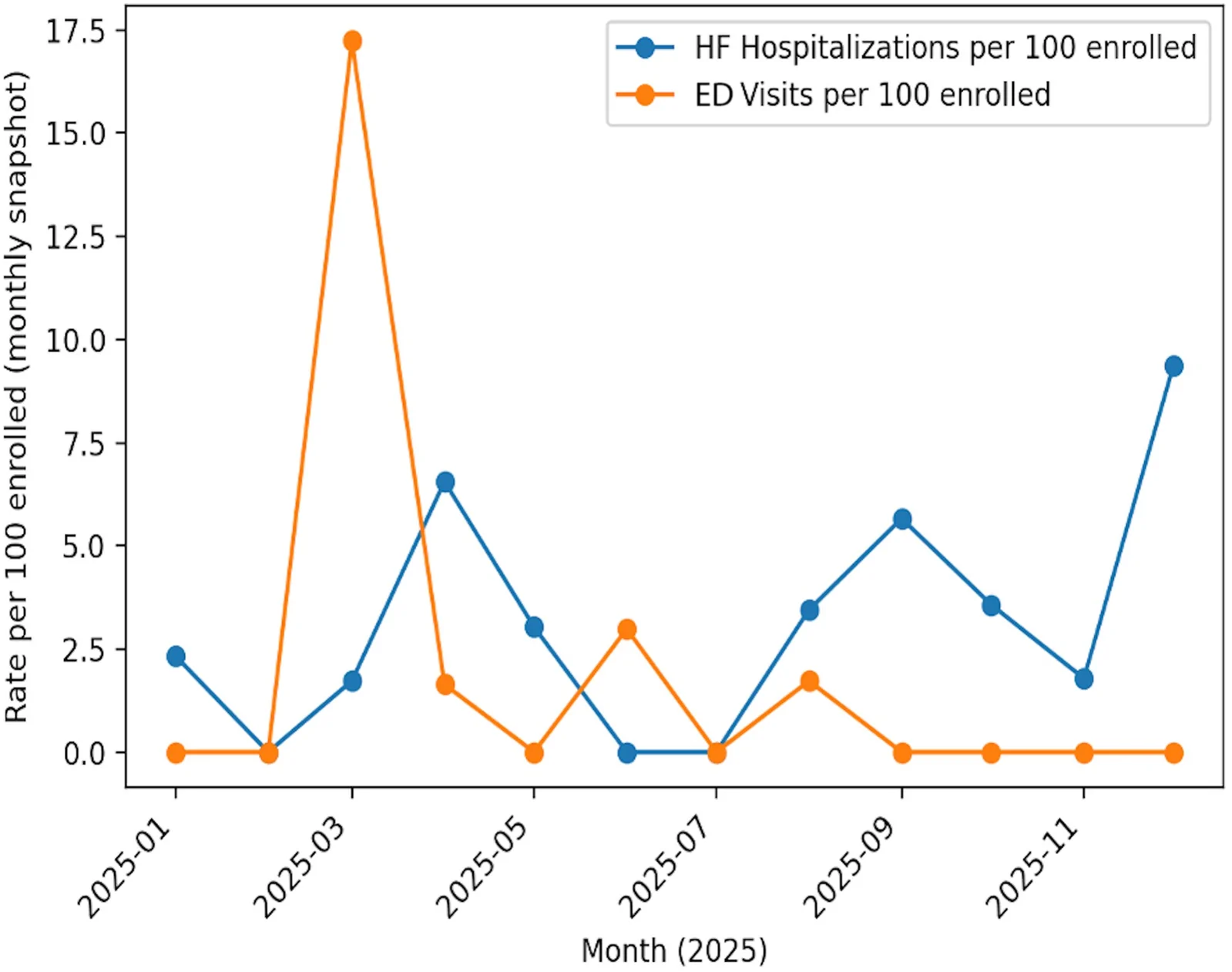
HR-Related Acute Care Utilization Over Time Monthly heart failure (HF) hospitalizations and HF-related emergency department (ED) visits, standardized per 100 enrolled patients, during the study period (2025).

### Outpatient diuretic optimization patterns

High-intensity outpatient oral diuretic optimization was consistently observed, with month-to-month variation. Oral diuretic adjustments ranged from approximately 25 to 100 events per 100 enrolled patients monthly and remained elevated after a midyear peak. In contrast, IV diuretic administration remained lower and relatively stable, ranging from approximately 5 to 21 events per 100 enrolled patients. Oral diuretic optimization remained the predominant strategy for outpatient congestion management (Figure 2).

**Figure 2:**
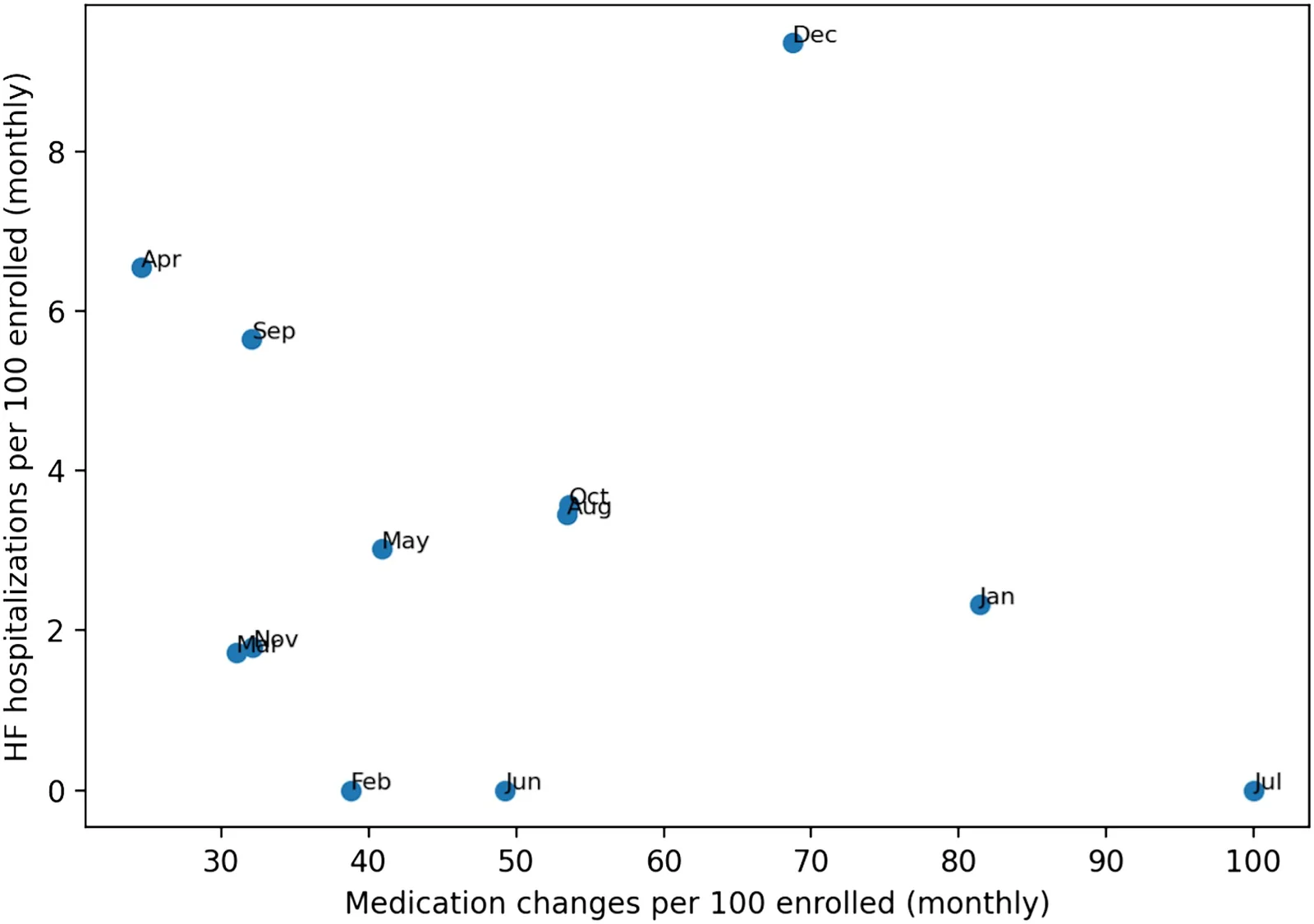
Outpatient Oral Diuretic Adjustment and Care Intensity Over Time Monthly standardized rates of education encounters, medication changes (oral diuretic adjustments for optimization), and intravenous diuretic administration per 100 enrolled patients during the study period (2025).

### Association between diuretic optimization and HF hospitalizations

Same-month analyses did not demonstrate a linear relationship between oral diuretic optimization intensity and HF hospitalizations, likely reflecting confounding by indication. In exploratory lagged analyses, higher outpatient oral diuretic optimization intensity in the preceding month was associated with fewer subsequent HF hospitalizations (incidence rate ratio: ≈0.94; *P* ≈ 0.046). Education encounters and IV diuretic (furosemide [Lasix, Hospira]) administration demonstrated favorable but nonsignificant associations (Figure 3).

**Figure 3:**
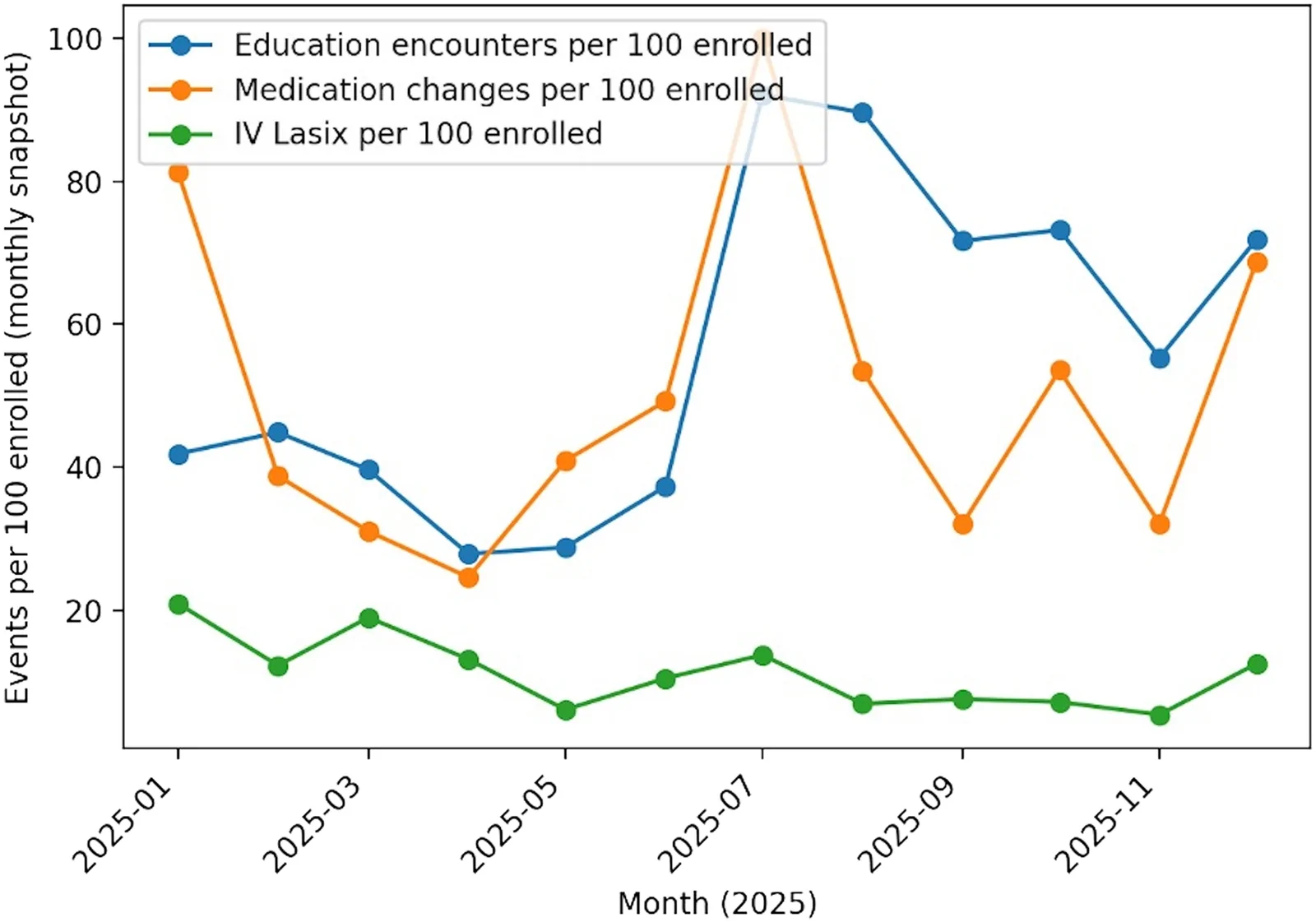
Outpatient Oral Diuretic Adjustment and HF-Related Hospitalizations Scatterplot depicting monthly medication changes (oral diuretic adjustments for optimization) and heart failure (HF) hospitalizations, standardized per 100 enrolled patients during the study period (2025). Each point represents a single month. IV = intravenous.

### Patient-level pre-post analysis

Among patients with complete data (n = 38), the proportion experiencing at least 1 HF hospitalization declined from 34.2% in the 3 months before enrollment to 18.4% in the 3 months following enrollment. These findings are descriptive and hypothesis generating.

### Impact and scalability

Stable HF hospitalization rates despite increasing enrollment suggest that proactive outpatient congestion management may be scalable without increasing acute care utilization. The relatively low use of IV diuretics further suggests that many episodes of worsening congestion were effectively managed with oral therapy alone.

### Future direction

Prospective multicenter studies using patient-level data, standardized congestion assessment, and remote monitoring are needed to determine whether proactive outpatient oral diuretic optimization causally reduces HF hospitalizations and improves long-term outcomes.

## Discussion

In this real-world observational evaluation, higher-intensity outpatient oral diuretic optimization within a structured HF program was associated with low and stable HF hospitalization rates despite increasing program enrollment. The CHF Navigator program emphasized early recognition of congestion and proactive oral diuretic adjustment, with relatively low reliance on outpatient IV diuretics. Lagged analyses demonstrated that greater oral diuretic optimization intensity was associated with fewer subsequent HF hospitalizations, supporting early outpatient intervention.

These findings extend prior evidence supporting structured outpatient HF management programs, including multidisciplinary and nurse-led models associated with reduced HF hospitalizations.^4^^,^^5^ Most prior studies have focused on GDMT titration, education, and self-care, with limited evaluation of the intensity of outpatient congestion management. Our analysis addresses this gap by longitudinally quantifying outpatient oral diuretic optimization and demonstrating its temporal association with HF hospitalization patterns.^7^

IV diuretic utilization remained comparatively low, suggesting that many episodes of worsening congestion were managed effectively with oral therapy. Whereas prior studies support outpatient IV diuretics and other resource-intensive strategies,^8^ these findings suggest that early clinic-driven oral diuretic optimization may represent a scalable and resource-efficient congestion management approach.

### Study Limitations

This retrospective observational analysis cannot establish causality and is subject to residual confounding. Diuretic dosing decisions were individualized and retrospectively extracted, limiting assessment of specific dosing strategies. Confounding by indication likely influenced same-month analyses, although lagged models were used to evaluate temporal relationships. Program-level analyses relied on aggregate data and could not fully adjust for patient-level characteristics. The patient-level pre-post analysis was limited by sample size and should be interpreted descriptively. Outcomes were restricted to acute care utilization, and all-cause utilization and mortality were not assessed.

## Conclusions

By quantifying outpatient oral diuretic optimization intensity and demonstrating its temporal association with subsequent HF hospitalization rates, this study supports a proactive, clinic-driven approach to management of congestion. Early outpatient oral diuretic optimization may reduce HF hospitalizations and represents a scalable strategy to complement GDMT and improve HF care delivery.

**Visual Summary:**
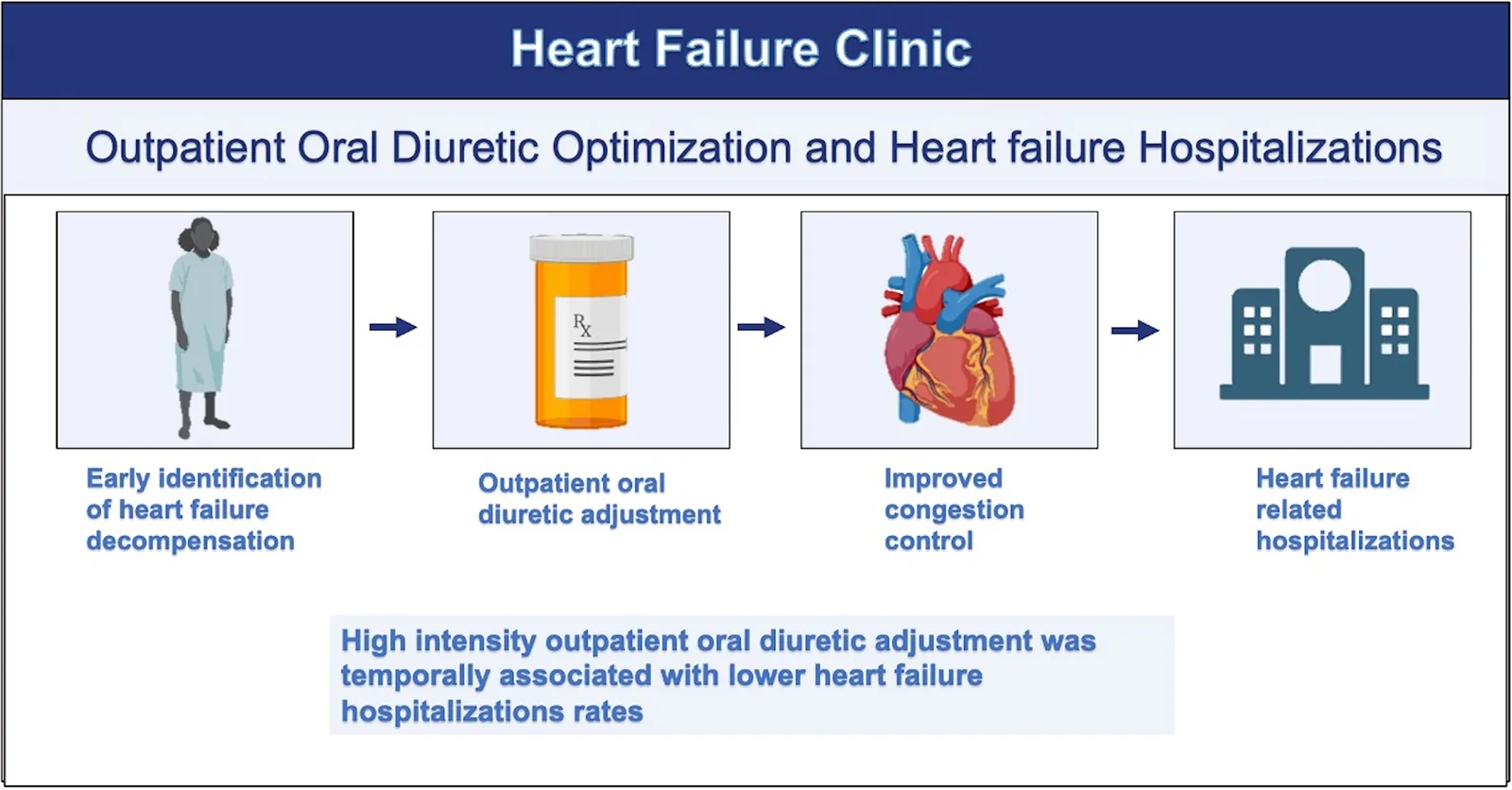
Higher-Intensity Outpatient Oral Diuretic Optimization Was Temporally Associated With Lower Heart Failure Hospitalization Rates

### Declaration of Generative AI and AI-Assisted Technologies

During the preparation of this work the authors used ChatGPT to assist with language editing and manuscript organization. After using this tool, the authors reviewed and edited the content as needed and take full responsibility for the content of the published article.

### Data Availability Statement

The data that support the findings of this study are not publicly available due to institutional restrictions and protection of patient privacy, but are available from the corresponding author on reasonable request.

**Take-Home Message**

• Higher-intensity outpatient oral diuretic optimization may represent a scalable early congestion management strategy associated with reduced HF hospitalizations.

## Funding Support and Author Disclosures

The authors have reported that they have no relationships relevant to the contents of this paper to disclose.
